# Effects of a One-Year Intensified Weight Loss Program on Body Composition Parameters in Patients with Severe Obesity and Obstructive Sleep Apnea (OSA): A Randomized Controlled Trial

**DOI:** 10.3390/nu16244255

**Published:** 2024-12-10

**Authors:** Laia Miralles-Llumà, Nuria Vilarrasa, Carmen Monasterio, Carla López-Padrós, Carolina Alves, Rosa Planas, Lorena Arribas, Monica Montserrat, Sandra Pérez-Ramos, Natàlia Pallarès, Neus Salord

**Affiliations:** 1 Multidisciplinary Sleep Unit, Department of Respiratory Medicine, Hospital Universitari de Bellvitge, L’Hospitalet de Llobregat, Feixa Llarga, s/n., 08907 Barcelona, Spain; 2 Department of Endocrinology and Nutrition, Hospital Universitari de Bellvitge, L’Hospitalet de Llobregat, 08907 Barcelona, Spain; 3 Section of Gastroenterology, Hepatology and Nutrition, Hospital Sant Joan de Déu, Esplugues de Llobregat, 08907 Barcelona, Spain; 4 Program PhD Nutrition and Food, University of Barcelona, L’Hospitalet de Llobregat, 08907 Barcelona, Spain; 5 Section of Endocrinology, Bellvitge Biomedical Research Institute (IDIBELL), L’Hospitalet de Llobregat, 08907 Barcelona, Spain; 6 CIBER de Diabetes y Enfermedades Metabólicas Asociadas (CIBERDEM), 28029 Madrid, Spain; 7 Section of Respiratory Medicine, Bellvitge Biomedical Research Institute (IDIBELL), L’Hospitalet de Llobregat, 08907 Barcelona, Spain; 8 Department of Rehabilitation, Hospital Universitari de Bellvitge, L’Hospitalet de Llobregat, 08907 Barcelona, Spain; 9 Clinical Nutrition Unit, Catalan Institute of Oncology (ICO), L’Hospitalet de Llobregat, 08907 Barcelona, Spain; 10 Section of Oncology, Bellvitge Biomedical Research Institute (IDIBELL), L’Hospitalet de Llobregat, 08907 Barcelona, Spain; 11 Biostatistics Unit, Bellvitge Biomedical Research Institute (IDIBELL), L’Hospitalet de Llobregat, 08907 Barcelona, Spain

**Keywords:** obstructive sleep apnea, visceral adipose tissue index, intramuscular adipose tissue index, obesity, weight loss, dietary/lifestyle intervention, Mediterranean diet

## Abstract

**Background:** Studies focusing on the effects of lifestyle strategies on patients with obstructive sleep apnea (OSA) that go beyond body weight and explore body composition are currently scarce and inconclusive. **Objectives/Methods:** The aim of this study was to evaluate the effects of a 12-month intensive life intervention program (ILI), based on a hypocaloric Mediterranean diet, on changes in the body composition parameters as assessed by abdominal computed tomography (CT) and the cardiorespiratory profile of patients with severe OSA and grade I–II obesity, compared to patients receiving standard care. Resultts:Thirty-four patients (30 males and four females) were randomly assigned to an intervention group (IG) (*n* = 18) or a control group (CG) (n = 16). We observed an improvement in OSA severity following the intervention. Patients in the IG lost 8.2% of their body weight compared to 0.1% of the patients in the CG (*p* < 0.001), and this loss was primarily due to reductions in total body fat, visceral adipose tissue index (VATI) [IG −19.4 (18.1) cm^2^/m^2^ versus CG 2.32 (11.6) cm^2^/m^2^, *p* < 0.001], and a tendency toward lower intramuscular adipose tissue index (IMATI) [IG −0.69 (0.85) cm^2^/m^2^ versus CG 0.04 (1.3) cm^2^/m^2^, *p* = 0.098]. These changes were associated with an improvement in patients’ metabolic and inflammatory profile. Younger age and a higher degree of obesity at baseline were associated with greater weight loss. Conslusions: In conclusion, the ILI was effective in reducing 8.2% of body weight at 12 months, leading to favorable changes in patients’ body composition profile that resulted in healthier metabolic and inflammatory parameters.

## 1. Introduction

Obesity, and central obesity in particular, is the main predictive factor for obstructive sleep apnea (OSA) [1,2,3]. Consequently, current clinical guidelines for OSA treatment include weight loss interventions with diet as a first non-pharmacological step strategy [4,5]. Previous studies have shown that a loss of 10% in initial body weight by means of intensified, structured weight loss programs is highly effective in improving OSA in the short and medium term [6,7,8,9,10]. Of note, in the Sleep Ahead Trial, patients randomized to an intensive lifestyle intervention (ILI) lost 10% of their initial body weight and were three times more likely to achieve total remission of OSA compared to patients receiving standard diabetes support and education [10]. However, these previous weight loss programs were highly heterogeneous, with some studies initially using a very-low-calorie diet (VLCD) with meal replacements, while others focused on caloric restriction, primarily through fat reduction and controlled carbohydrate intake. In most of these studies, physical activity was unsupervised. In a previous study by our group, a year-long intensified weight loss program based on the Mediterranean diet in patients with severe OSA and grade I-II obesity proved superior to standard treatment, providing a 8.2% loss in body weight and a notable improvement in OSA severity, allowing for the withdrawal of continuous positive airway pressure (CPAP) in 28% of patients [11]. The longest longitudinal study to date is the extension of the Sleep Ahead trial, which demonstrated the persistence of its benefits, maintaining OSA remission rates at 10 years in 34.4% of the ILI patients compared to 22% of the patients receiving standard diabetes support and education. The overall reduction in OSA severity was related to changes in body weight, the baseline apnea hypopnea index (AHI), and the intervention arm, independent of weight change. This suggests that factors associated with ILI other than weight loss per se may have contributed to an improvement in OSA severity [12]. In this sense, new radiological imaging methods are increasingly used to determine body composition parameters, providing a detailed, near-anatomical visualization of tissues such as body fat and accurate information about their spatial distribution [13]. This body composition approach may better explain improvements beyond weight loss alone.

Our current knowledge of the physiopathology of obesity suggests that it is not only excess body weight but also increased ectopic adipose tissue deposits in multiple organs that are the key determinants of the complications of obesity and associated diseases. In this sense, increased deposits of visceral adipose tissue (VAT) are closely linked to systemic inflammation and metabolic disorders, and this more centrally deposited fat is particularly closely related to OSA [14]. Moreover, increased deposits of intramuscular adipose tissue (IMAT) have been associated with a deterioration in muscle function, contributing to the development of sarcopenia, which is implicated in the progression of chronic diseases including OSA [15,16,17,18]. However, to date, there is a lack of data regarding changes in ectopic adipose tissue deposits in patients with OSA before and after weight loss interventions. One of the main limitations is that this requires radiological techniques different from more accessible methods like dual-energy X-ray absorptiometry (DXA) and bioelectrical impedance analysis (BIA). Among these techniques, computed tomography (CT) plays a special role as it is uncomplicated, often part of routine radiological examinations, and offers advantages over magnetic resonance imaging due to its standardized scale for measurements (Hounsfield units) [19].

Against this background, the aim of our study was to evaluate the changes in body composition parameters such as adipose tissue, including IMAT, VAT, and subcutaneous adipose tissue (SAT), as well as skeletal muscle index (SMI) and muscle attenuation (MA) using CT scan images taken after a 12-month intensive weight loss program involving a hypocaloric Mediterranean diet in patients with severe OSA and grade I–II obesity compared to patients receiving standard care. We also analyzed the impact of macronutrient intake on body composition and cardiovascular risk factors and their direct effect on respiratory parameters.

## 2. Materials and Methods

### 2.1. Study Design

We designed a randomized, controlled, non-blinded, parallel-group prospective clinical trial [11]. The study aimed to test the hypothesis that a 12-month intensive weight loss program applied to patients with severe OSA undergoing continuous positive air pressure (CPAP) treatment can achieve sufficient weight loss to improve OSA severity and metabolic dysfunction. The study protocol was approved by the local ethics committee (PR209/14. Approval Date: 6 November 2014; Approved affiliation: Bellvitge University Hospital) and its registration number is NCT02832414 on the clinicaltrial.gov website. The primary outcomes of the study have been published [11]. The present paper is based on a secondary endpoint that consisted of analyzing the changes in body composition parameters, cardiovascular profile, and the impact of macronutrient intake in the intervention group (IG) compared to the control group (CG).

### 2.2. Participants

Potential candidates for the study were recruited from the Sleep Unit at the Bellvitge University Hospital outpatients clinic. The recruitment period started in November 2014 and ended in April 2016. Candidates were considered eligible for participation in the study if they were adults (25–60 years old) with class I and II obesity (body mass index (BMI) 30–40 kg/m^2^), severe OSA (AHI > 30 events/h), and had been undergoing treatment with CPAP for a minimum of six months before inclusion. Candidates were excluded on the basis of the following criteria: (a) physical activity or dietary contraindications; (b) cognitive impairment or psychiatric disorders that impeded patients’ understanding of the program; (c) severe diseases; (d) major cardiovascular disease; (e) clinical instability within the previous month; (f) prior bariatric surgery; (g) refusal to participate in the study; and (h) participation in another clinical trial. Eligible candidates were informed of the aims and procedures of the study and signed a written consent form for participation.

### 2.3. Randomization

Enrolled participants were randomized to one of the two groups for 12 months: the non-intervention group (receiving standard care recommendations) or the intervention arm undergoing an intensive weight loss program. Randomization was performed using an automatic computer-generated program that incorporated block randomization with a block size of five.

### 2.4. Procedures and Measurements

Anthropometric variables, including body weight and height, waist, and neck circumference, were measured following standard procedures. As described previously [11], all patients underwent a polysomnography (PSG) at baseline, and at 3 and 12 months. Patients were instructed to stop CPAP treatment for the three nights prior to the polysomnography.

An abdominal computed tomography (CT) scan was performed at baseline and at 12 months. A single CT image of the third lumbar vertebra (L3) was selected on the axial cross-section CT component as a reference point, based on previous reports [20]. The Standardized Hounsfield Unit (HU) scale was used to quantify body compartments including skeletal muscle from −29 to +150 HU, VAT from −150 to −50 HU, SAT from −190 to −30 HU, and IMAT from −190 to −30 HU using SliceOmatic© software (v5.0 Rev 8, Tomovision, Magog, QC, Canada). Cross-sectional areas were then normalized for height and reported as indexes (cm^2^/m^2^): skeletal muscle index (SMI), visceral adipose tissue index (VATI), subcutaneous adipose tissue index (SATI), and intramuscular adipose tissue index (IMATI). Mean muscle attenuation (MA) in HU was reported for the entire muscle area at the third lumbar vertebra. Sarcopenia was defined as per Martin et al. [21] in individuals with overweight and obesity as SMI < 53 cm^2^/m^2^ in men or <41 cm^2^/m^2^ in women. 

To assess cardiometabolic parameters, the morning after the PSG (8–9 a.m.), a venous blood sample was obtained from all patients in fasting conditions. Fasting blood glucose (FBG), total cholesterol, triglycerides, and high-density lipoprotein (HDL) levels were determined by molecular absorption spectrometry in the Cobas c702 analyzer (Roche Diagnostics, Basel, Switzerland). Low-density lipoprotein (LDL) cholesterol and very low-density lipoprotein (VLDL) cholesterol were determined by the Friedewald Formula. Glycosylated hemoglobin was determined by high-pressure cation-exchange liquid chromatography in the HA-AutoA1C 8180 analyzer (Arkray Kyoto, Japan). C-reactive protein was determined by Immunoturbidimetry in the Cobas c702 analyzer (Roche Diagnostics, Basel, Switzerland). Only one measurement was performed for each determination. Clinical blood pressure was measured by using a standard sphygmomanometer while the subject was seated at rest and was taken as the mean value of at least two measurements separated by five minutes, and an additional measurement was made if there was a difference of >5 mmHg between the two.

OSA-related symptoms and CPAP compliance by the mean hours of usage per night were recorded by the time counter.

The primary data collection time points were at 3 and 12 months. The 3-month time point marked the end of the intensive intervention phase, which included a two-week very-low-calorie diet with meal replacements, followed by a less intensive hypocaloric diet with continued meal replacement use, assessing short-term outcomes, while 12 months was used to evaluate long-term outcomes.

### 2.5. Intensive Weight Loss Program

The program consisted of a three-phase, progressive hypocaloric diet: During the initial phase, over a 15-day period, patients followed a very low-calorie diet (VLCD) of 600–800 kcal per day, adjusted for sex and physical activity level. Low-calorie liquid meal replacements (Optifast^®^ [Nestle Health Science, Vevey, Switzerland]) were consumed 3–4 times daily, replacing the three main meals (breakfast, lunch, and dinner). The second phase consisted of a 1200 kcal diet replacing dinner with a low-calorie liquid meal (Optifast^®^ [Nestle Health Science, Vevey, Switzerland], accompanied by the initiation of physical activity. Patients also attended biweekly dietary consultations with a specialized dietitian, the first two of which were group-based, followed by a combination of individual and alternating group sessions. Each session lasted 60–90 min, was supervised by the study dietitian, and focused on building group support, offering motivation, and providing dietary counseling and education. In the third phase, patients followed a 1200–1800 kcal Mediterranean diet with a caloric restriction of 500–700 kcal based on individual basal metabolic requirements calculated using the Mifflin-St Jeor equation. The resting metabolic rate resulting from this calculation was multiplied by an activity-level factor: sedentary (×1.2), lightly active (×1.375), moderately active (×1.55), active (×1.725), and very active (×1.9). Patients were encouraged to follow a hypocaloric Mediterranean diet with a total fat intake not exceeding 30% of their daily caloric intake, primarily derived from vegetable sources (e.g., virgin olive oil and nuts). Protein sources emphasized plant-based foods (e.g., legumes) and lean animal sources (e.g., fish or poultry), with a limited consumption of red and processed meats. Carbohydrate intake was encouraged to come from minimally processed, fiber-rich foods with a low glycemic index, such as vegetables, fruits, and whole grains [22].

Once the VLCD phase had finished, unsupervised physical activity was introduced. Patients were advised to exercise from three to five days a week to achieve the goal of 150 min per week, in line with Word Health Organization (WHO) recommendations [23]. It was advised that every workout session should consist of stretching, 5–10′ of warm up (50–60% of maximum heart rate (HR)), 40–50′ of aerobic exercise (70–80% of maximum HR), and 5–10′ of cool down (50–60% of maximum HR). At each individual visit, a questionnaire was performed asking about the frequency, duration, and intensity of physical exercise. The rehabilitation service provided care to patients who presented musculoskeletal complaints during the exercise program (a visual analog scale pain score ≥ 4). 

The CG participants were provided with general oral and written information about a healthy diet and exercise at baseline as standard care.

#### Estimation of Nutrient Intake

Dietary consultations were conducted biweekly with a specialized dietitian; the first two sessions were group-based, followed by a combination of individual and alternating group sessions. Consultations were then reduced to monthly from months three to six, and quarterly from months six to 12. To monitor adherence, blood β-hydroxybutyrate was used to assess individual fat metabolism during a low-calorie diet. Nutritional ketosis was defined as blood ketone levels between 0.5 and 3 mmol/L, which also represents the optimal ketone range for weight loss. Ketonemia test strips and 24 h recalls were conducted by the dietitian during the first three months [24]. The 24 h recalls were used to obtain more stable macronutrient estimates, as noted by Bailey [25]. A 3-day self-reported food record was used from the third month until the end of the study for clinical follow-up. This 3-day food record was analyzed at baseline, 3, and 12 months. Participants were asked to report the quantities of the individual foods and beverages consumed using common household measures or typical portion sizes. The registers were analyzed by an expert dietitian using the PCN Pro 1.0 program.

### 2.6. Statistical Analysis

The primary endpoint of the ancillary study was a reduction in the rate of apnea–hypopneas/hour at 12 months compared to baseline [11]. The present study is based on a secondary endpoint that consisted in studying the effects of a 12-month ILI program, based on a hypocaloric Mediterranean diet, on changes in body composition parameters assessed using abdominal computed tomography (CT) and the cardiorespiratory profile of patients with severe OSA.

The study size was calculated according to the ancillary study, whose main variable was the reduction in subjects’ final AHI with respect to baseline. Accepting an alpha risk of 0.05 and a power of 80% in a bilateral approach, a final sample of 42 patients was deemed sufficient to detect a difference of 15 points in the AHI between the CG and IG (a clinically relevant difference), considering a standard deviation (SD) of 15 and a loss of 25%. Balanced groups were estimated [11].

Categorical variables are presented as the number of cases and percentages, and continuous variables are presented as mean and (SD) or median and interquartile rank [IQR]. Continuous variables were compared using the Student’s *t*-test or the Mann–Whitney U-test, as appropriate. Fisher’s exact test or Pearson’s χ^2^ test were used to assess the relationship between categorical variables. We studied weight loss and body composition parameter correlations with different macronutrient changes using Pearson’s correlation coefficient. To identify the factors associated with weight loss over time, we estimated a linear mixed-effects model using the lme4 package for R 4.0 [26,27]. This method accounts for clustered data within the same subject, using repeated weight loss measurements over time. To show the magnitude of the association, we reported regression coefficients corresponding to the fixed effects and their corresponding 95% confidence intervals, and *p*-values. All model assumptions were assessed graphically and analytically. The adjusting variables considered were treatment group, age, baseline BMI, and baseline waist circumference. Analyses were performed using R software 4.0 [26,27]. The level of statistical significance was set at 5%.

## 3. Results

**Figure 1:**
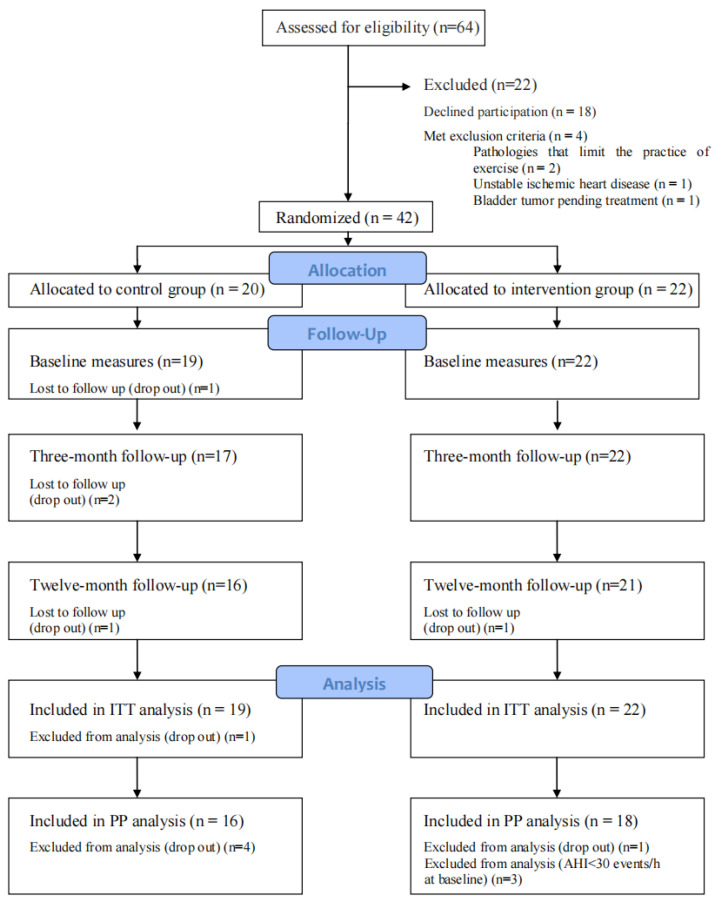
Study flowchart. CG: control group; IG: intervention group.

A total of 42 patients (38 males and four females) were randomized to the IG (n = 22) or CG (n = 20) groups. Patients had a mean baseline BMI of 35 (2.7) kg/m^2^ and a mean AHI of 69 (20) events/h. Four patients from the control group left the study, while only one patient in the intervention group left. Three patients from the intervention group had an AHI below 30 in the baseline polysomnography of the trial and were therefore not included in the per-protocol analysis. There were no differences regarding the main characteristics between per-protocol and intention-to-treat analyses; therefore, the data shown are from the per-protocol analysis, for which all data are available (Figure 1).

The baseline characteristics of the participants are shown in Table 1. Patients were broadly similar regarding sex, age, and BMI. However, patients in the CG had a higher waist circumference and a tendency toward having a higher weight, estimated fat mass, and VAT. (Table 1). No differences were found regarding comorbidities between the two groups (Table S1). Hypertension was the most prevalent comorbidity, followed by dyslipidemia and type 2 diabetes mellitus (T2DM). There were no differences between the two treatment groups in AHI (Table 1). Similar baseline glucose, glycosylated hemoglobin (HbA_1c_), lipid profile, and CRP were observed between groups (Table S2).

### 3.1. Baseline Nutritional Parameters

There was no difference in diet composition between the groups at baseline; however, the CG patients consumed more alcohol (gr) than IG patients (Table 2 and Table S1). The analysis of the dietary records showed a similar baseline total energy intake and similar distribution in macronutrients, total carbohydrates (39.9 vs. 41.8%), proteins (16.3 vs. 15.9%), and total lipids (39.9 vs. 41.4%). A predominance of monounsaturated fatty acid intake (19.6 vs. 19.8%), followed by saturated fatty acids (10.9% vs. 12.2%) and polyunsaturated fatty acids (6.2% vs. 5.9%), was similarly observed in both groups. Table 2 shows the baseline diet composition in grams and Table S2 represents the distribution of calories in macronutrients.

### 3.2. Changes in Anthropometry

**Figure 2:**
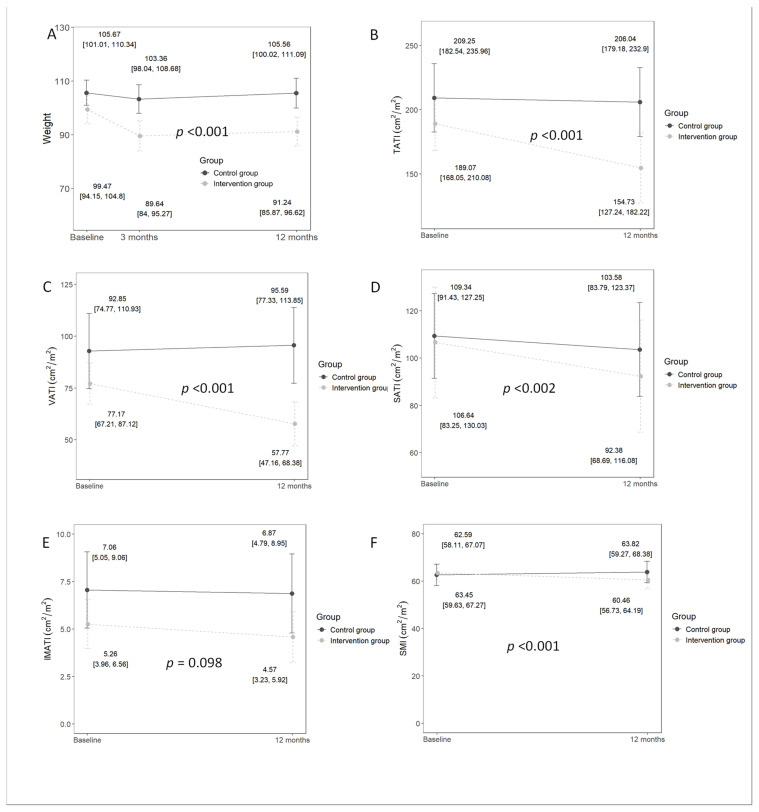
Change in weight and abdominal CT scan main variables. (**A**) Change in weight over the follow-up period. (**B**) Change in TATI over the follow-up period. (**C**) Change in VATI over the follow-up period. (**D**) Change in SATI over the follow-up period. (**E**) Change in IMATI over the follow-up period. (**F**) Change in SMI over the follow-up period. The mean values are represented by dots, and the values in square brackets represent the confidence intervals. The *p*-value indicates the significance of the difference between groups. TATI: total adipose tissue index; VATI: visceral adipose tissue index; SATI: subcutaneous adipose tissue index; IMATI: intramuscular adipose tissue index; SMI: skeletal muscle index.

Between baseline and three months, patients belonging to the CG lost −2.3 (4.3) kg of body weight compared to −10.5 (3.7) kg in the IG (*p* < 0.001), representing a total weight loss of 2.2% in the CG versus 10.6% (*p* < 0.001) in the IG. However, at 12 months, a small weight regain was observed in both groups, resulting in a total weight change of −0.1 (4.8) kg in the CG and −8.2 (5.9) kg in the IG (*p* < 0.001), (Figure 2A), corresponding to a total weight loss of 0.15% in the CG compared to 8.2% in the IG (*p* < 0.001). There were reductions in BMI −0.07 (1.5) Kg/m^2^ vs. −2.8 (1.9) Kg/m^2^ (*p* < 0.001), neck circumference 0.0 (1.6) cm vs. −2.2 (1.4) cm (*p* = 0.001), and waist circumference −1.13 (5.25) vs. −7.29 (6.11) cm (*p* = 0.005) in favor of the IG at 12 months.

The body composition analysis of abdominal CT scan images showed a higher reduction in VATI, SATI, and TATI in the IG compared to the CG; IMATI reduction did not reach statistical significance (Figure 2B–E, respectively). Although there was a significant reduction in SMI of 0.50 (2.11) cm^2^/m^2^ vs. −3.00 (2.46) cm^2^/m^2^ (*p* < 0.001) between the CG and IG, respectively, it was not clinically relevant, maintaining a non-significant difference in muscle attenuation: −0.21 (1.31) HU vs. −0.31 (1.75) HU (*p* = 0.861). Therefore, in the IG, the greatest reductions were in TATI and VATI, with reductions of 19.4% and 24.3%, respectively, compared to a 4.7% reduction in SMI. Conversely, in the CG, there was an increase in TATI, VATI, and SMI of 1.79%, 4.3%, and 0.8%, respectively. Sarcopenia was not present at baseline in any patient of the IG, and, at the end of the study, only one patient developed it.

### 3.3. Adherence to Diet and Physical Exercise Program

During the first two weeks of the very-low-calorie diet, ketone levels were positive in all IG patients. During the following ten weeks, all patients in the IG maintained a restriction of 500–700 Kcal according to their 3-day food records compared with two patients in the CG. During the last nine months, only 8 (44.4%) patients maintained a restriction of 500–700 Kcal in the IG compared to two (12.5%) patients in the control group. Regarding physical exercise, at three months only seven (43.8%) patients from the IG met the goal of >150 min of physical exercise per week, decreasing to three (17.6%)) patients at 12 months. On the other hand, five patients (33.3%) from the control group practiced >150 min of physical exercise per week at three months, decreasing to four patients (26.7%) at 12 months.

### 3.4. Changes in Diet Composition

At three months, there was only a significant calorie content reduction in the IG at the expense of fats, concretely due to a reduction in saturated, polyunsaturated, and monounsaturated fats (Table 2). At 12 months, as expected, there was a slight increase in energy consumption in the IG compared with the more hypocaloric 12-week period. Overall, compared to baseline values and superior to the CG, the IG experienced a significant decrease in energy intake and in the consumption of carbohydrates and saturated and monounsaturated fats (Table 2). Changes in kcal distribution in macronutrients are shown in Tables S2 and S5.

### 3.5. Changes in Metabolic and Blood Pressure Parameters

In terms of metabolic improvement, there was a significant increase in high-density lipoprotein cholesterol and a notable decrease in HbA1c in the IG compared to the CG. We only observed an improvement in fasting glucose in the IG compared to the CG at the three-month visit (Table 3). There was a significant improvement in CRP at 12 months in the IG. No changes in systolic and diastolic blood pressure were observed throughout the study.

### 3.6. Changes in Obstructive Apnea

As published previously [11], a non-significant improvement in AHI was observed in the IG compared to the CG (Table S4). Regarding OSA severity, using AHI cut offs for mild, moderate, and severe obstructive sleep apnea, at 12 months, one patient in the intervention group had improved by two categories and four patients had improved by one. No one in the control group had improved their OSA category at 12 months. As a result, 28% of patients in the treatment group changed from severe OSA to mild-moderate, compared to none in the control group (*p* = 0.046, Fisher’s exact test).

### 3.7. Correlations Between Body Composition Changes and Dietary Composition Changes

Changes in body weight correlated significantly with variations in diet energy content (Kcal) (r = 0.46, *p* = 0.007), lipid content (g) (r = 0.381, *p* = 0.029), and carbohydrate content (g) (r = 0.437, *p* = 0.011).

A tendency was observed towards a correlation with changes in energy intake and changes in IMATI (r = 0.33, *p* = 0.075), estimated fat mass (r = 0.338, *p* = 0.068), SATI (r = 0.356, *p* = 0.053), TATI (r = 0.326, *p* = 0.079), and MA (r = 0.335, *p* = 0.071).

A tendency was observed towards a correlation of changes in lipid intake (g) with IMATI (r = 0.311, *p* = 0.094). Changes in carbohydrate intake (g) correlated positively with SATI (r = 0.382, *p* = 0.037) and MA (r = 0.366, *p* = 0.047). We were unable to find a correlation between protein intake and lean mass, SMI, or MA.

### 3.8. Correlations Between Biochemical Parameters and Body Composition Changes

Changes in HDL-c correlated negatively with VATI (r = −0.404, *p* = 0.03) and there was a tendency towards a negative correlation between changes in HDL-c and TATI (r = −0.366, *p* = 0.051) and estimated fat mass (r = −0.349, *p* = 0.063).

### 3.9. Correlations Between Respiratory Parameters and Body Composition Changes

No correlation was found between AHI change and changes in macronutrients or body composition parameters.

### 3.10. Correlations Between Metabolic and Blood Presure Changes and Dietary Composition Changes

Changes in lipids (g) correlated with changes in HbA_1_c (r = −0.359, *p* = 0.031). Changes in polyunsaturated fat (g) (g) correlated with changes in diastolic and systolic blood pressure (r = 0.469, *p* < 0.006) (r = 0.4, *p* < 0.020). No other correlation was found between blood pressure changes and other metabolic and cardiovascular risk factors and changes in macronutrients.

### 3.11. Mixed-Effects Models Estimating Change in Weight, Anthropometric and Body Composition over Time

To compare the value of weight over time at the baseline visit, at 3 months, and at 12 months, we fitted a mixed-effects model adjusted for potential confounders. The change in weight (outcome variable) adjusted by age, baseline waist circumference, and baseline BMI was related to the intervention, changes in energy intake, and changes in lipids (g). On average, patients in the IG had lost seven kilograms at the 3-month and eight at the 12-month visits. In the control group, the difference in weight loss was not significant. For each SD of decrease in Kcal with respect to the mean value of the Kcal difference, patients lost two kilograms of weight. For each SD of decrease in lipids (g) intake, patients had lost two kilograms at the three-month visit and three at the 12-month visit. No effect was observed regarding carbohydrates or protein change (g) on weight change. Younger age and greater baseline BMI were associated with greater weight loss in the intervention mixed-effects model (Table 4). Meanwhile, greater baseline BMI and a greater baseline waist circumference were associated with greater weight loss in the models adjusted by energy intake changes and by lipid changes (Table 4).

Additionally, linear models were used to explore the impact of macronutrient changes on anthropometry and body composition parameters. The change over time in IMAT adjusted by age, baseline waist circumference, baseline BMI, and baseline IMAT was only related to changes in energy intake (Tables S6 and S7).

## 4. Discussion

To the best of our knowledge, this is the first attempt to explore the impact of an annual intensive weight loss program on body composition using an advanced analysis of abdominal CT scan images in patients with grade I–II obesity and severe OSA undergoing CPAP treatment. These results complement that of the ancillary study [11] in which we demonstrated an improvement in OSA severity following an intensive weight loss program based on a hypocaloric Mediterranean diet. In our present study, we found that patients randomized to this intensive intervention had improved body composition parameters including VATI and SATI. These changes were associated with an improvement in their metabolic and inflammatory profile. When controlled by confounders, weight loss was mainly explained by a calorie and lipid intake reduction. IMATI improvement was also explained only by calorie restrictions. Younger patients and those with a higher degree of obesity experienced greater weight loss.

Obesity contributes to the development of OSA mainly through fat accumulation around the neck and upper airway, which reduces the diameter of the airway and increases resistance to airflow, favoring airway collapse. Additionally, increased abdominal visceral adiposity decreases lung volumes, including functional residual capacity, and reduces traction on the pharynx, leading to increased pharyngeal collapsibility and, consequently, OSA [28]. With advances in body composition assessing technologies, a close relationship between OSA and abdominal visceral fat has been described in several studies [1,29]. Therefore, when addressing OSA treatment, not only body weight but also visceral adiposity should be taken into account.

Clinical guidelines for OSA treatment strongly recommended lifestyle change programs based on diet and exercise for its management [4]. Different weight loss interventions in this disease have been described in the literature, in which a loss of 10% or more of initial body weight led to an improvement in OSA severity [10,29,30,31]. Of all the possible dietetic approaches, the Mediterranean diet is most closely associated with a reduction in cardiovascular risks [32,33]. In this sense, some previous studies using a hypocaloric Mediterranean diet to treat OSA have demonstrated not only improvements in body weight, AHI, and OSA severity but also in metabolic profile (HDL-c and HbA1c) [34].

A limited number of studies have explored in depth changes in body composition parameters following a dietary intervention in the setting of OSA. In a previous shorter RCT, the INTERAPNEA Study [35], adherence to an eight-week dietary intervention based on the Harvard Plate model plus moderate aerobic exercise, smoking cessation, alcohol intake avoidance, and sleep hygiene performed in men with severe OSA showed a body weight decrease of 7%, a decrease in fat mass assessed with DXA of 19%, and a VAT decrease of 26%, along with improvements in OSA severity (−23.8 events/h) and cardiometabolic risk (including blood pressure and glucose and lipid metabolism) [35]. In the Sleeping well trial [36], a 6-month lifestyle intervention based on a 5-day intermittent energy restriction regimen consisting of reduced energy intake (6300–7500 kJ/day) and two days between 2200 and 2760 kJ/day, a reduction in fat mass and VAT evaluated with DXA was observed in male participants. These regional body composition changes in males were positively associated with improvements in OSA severity (*p* < 0.01).

In our study, following a hypocaloric Mediterranean diet for 12 months proved to be a healthy approach, resulting in a significant reduction in VATI, SATI, and a tendency toward a reduction in IMATI in patients with OSA. To our knowledge, this is the first study to analyze all these parameters in the setting of a RCT with an intervention lifestyle program in patients with OSA and grade I–II obesity. Moreover, in our study, an improvement in lipid profile, glucose homeostasis, and chronic inflammation was observed in the intervention group. In line with our findings, in the PREDIMED plus trial [37], which included patients with metabolic syndrome and overweight or obesity randomized to a 3-year low-calorie Mediterranean diet compared to standard care, a reduction in DXA fat mass of −0.52 vs. −0.14%, in VAT of −72.2 vs. −5.50 g favoring the IG, together with a preservation of lean mass of 0.47 vs. 0.13%, respectively, was observed. In our study, greater fat mass and VAT loss was observed, probably because of the more intensified initial hypocaloric phase and the shorter duration of the trial.

The fact that, in our series, IMATI experienced a clinically relevant decrease in the intervention group is a highly outstanding finding. IMAT is a unique adipose depot that is closely associated with an increased risk of insulin resistance, subclinical atherosclerosis, metabolic syndrome, and cardiovascular disease [17,18,37,38,39,40,41]. IMAT can only now be quantified following advances in quantitative magnetic resonance imaging (MRI) and abdominal CT. It is an indicator of muscle fat infiltration that not only correlates with overall body fatness but also with lower muscle strength [42]. Weight loss is usually associated with an undesired loss of lean mass, similar to the process of aging which can lead to sarcopenia (loss of muscle and strength), leading to a reduction in energy expenditure and a potentially increased risk of weight regain in the midterm [43].

Although in our study the overall loss of SMI was statistically significant in the IG, the absolute reduction of −3.00 (2.46) units of cm^2^/m^2^ is not clinically relevant as it represents a total reduction of 4.7% and is outweighed by the benefit of the overall reduction in all adipose tissue compartments. In fact, only one patient developed sarcopenia as defined by Martin et al. [21] after the intervention. The current sarcopenic obesity (SO) consensus recommends not only the study of total muscle mass but also its function with dynamic tests [44]. In the EPISONO study [16], SO, defined as low appendicular skeletal muscle mass adjusted for BMI, but not obesity alone, was directly associated with OSA. Similarly, in a recent study analyzing the NHANES cohort from 2015 to 2018, a higher prevalence of SO, reaching 10.3%, was observed among participants with OSA compared to 4% in those without OSA. OSA was found to be a significant independent risk factor for SO by inducing muscle loss through unhealthy diet habits, higher BMI, chronic inflammation, insulin resistance, and low vitamin D [45]. It has been hypothesized that intermittent hypoxia in OSA can induce the activation of the hypothalamic–pituitary–adrenal axis, promoting muscle catabolism [46], which on its own can promote airway collapsibility, further worsening OSA. Therefore, in light of this evidence, lifestyle interventions for weight loss in patients with OSA should aim to integrate visceral fat and IMAT loss with the preservation of skeletal muscle mass to prevent sarcopenia.

Exercise adherence may impact weight loss and improvements in body composition. Increasing physical activity provides comprehensive health benefits and reduces the mortality rate associated with any cause [47]. Among different types of exercise, aerobic training is recommended for individuals with overweight, as it reduces body weight and fat mass and can improve cardio-metabolic markers [48]. Strength (resistance) training shows similar benefits [49], while combined (resistance + aerobic) training has been reported to yield more favorable effects on cardiometabolic markers compared to either resistance or aerobic training alone [50]. A recent meta-analysis [51] confirmed that three different exercise modalities (resistance, strength, and combined training) are effective in improving cardiometabolic parameters in individuals with overweight. In fact, a previous randomized study demonstrated that a hypocaloric diet without an exercise prescription is sufficient for short-term weight loss, but exercise plays a more significant role in improving body composition and functional capacity [52]. Body composition is more positively affected when individuals with overweight engage in moderate exercise (e.g., walking) compared to a hypocaloric diet alone. However, combined exercise, including both resistance and strength training, is the most effective protocol for maintaining body composition and muscle mass [52]. Additionally, some studies have demonstrated that resistance training plays a key role in mitigating the effects of sarcopenia [53]. In our study, we included a non-supervised moderate exercise regimen, as recommended by the WHO [23], which could contribute to weight loss and body composition improvement. However, it appears to be less effective than a combination of resistance and strength training. Moreover, the unsupervised exercise program resulted in very low compliance, making it difficult to assess its overall impact on body composition.

In our study, we identified which changes in macronutrients were more closely related to body composition parameters. We observed a tendency suggesting that reduced total energy and lipid intake was associated with reductions in VAT, SATI, and IMATI. IMATI remained the main determinant in the linear regression analysis. Our results are in accordance with a systematic review and metanalysis that demonstrated that high-fat diets significantly increased intramuscular fat deposits [54]. Although we were unable to find a correlation with a specific type of fat, as proposed by the Mediterranean diet, a reduction in saturated fats should target a greater proportion of mono and polyunsaturated fats. Of note, carbohydrate intake was associated with SAT, but this association was not sufficiently robust as it lost significance in the linear regression analysis. We were not able to find a correlation between protein intake and SMI or muscle attenuation. In fact, a non-clinically relevant decrease in protein intake was observed in our IG, but the percentage remained unaltered. It is known that a sufficient protein intake (25–30 g of protein per meal) is important for optimizing muscle protein synthetic response, but protein intake should be combined with resistance exercise. In our study, low–moderate exercise according to the WHO guidelines was indicated in the IG but it was self-applied by patients and, unfortunately, exercise was not accurately recorded.

As expected, HDL-c correlated negatively with TATI. These findings support a routine screening of the lipid profile and glucose metabolism as part of metabolic syndrome in patients with OSA and obesity.

Our dietetic approach for the IG was very successful, and 28% of patients in this group significantly improved their OSA severity and CPAP treatment could be removed. However, we were unable to find a correlation between AHI and changes in macronutrients or body composition parameters. The low number of patients could explain this lack of association.

Our study has several limitations. One important limitation relates to the study design and sample size, as this was a single-center clinical trial with a specific and relatively homogenous patient population. Participants included individuals with grade I–II obesity and severe obstructive sleep apnea (OSA). As a result, the findings may not be generalizable to patients with different characteristics, such as those with mild or moderate OSA, varying degrees of obesity, or additional comorbid conditions. Furthermore, the single-center nature of this study may limit its external validity. Another important limitation of the study was that the exercise regimen was unsupervised and was monitored solely through questionnaires assessing the frequency, duration, and intensity of activity, which led to low adherence to the exercise program. No objective measurements, such as actimetry, were used. Additionally, we did not use handgrip or dynamic tests to measure muscle strength. These tests could provide valuable insights into strength losses that may accompany muscle loss caused by the diet. As a result, the study’s conclusions regarding body composition and muscle preservation may be considered incomplete. Finally, consistent with the lower prevalence of OSA in women, a smaller number of female participants were recruited for the study. While changes in weight and fat distribution may differ between women and men, we were unable to analyze these differences separately. Finally, only C-reactive protein was analyzed as a proinflammatory marker; no other inflammatory markers were measured. Future studies could include additional markers, such as TNF-α receptors (R1 and R2), IL-6, MCP-1 (monocyte chemoattractant protein-1), leptin, and adiponectin (as an anti-inflammatory marker), or the leptin/adiponectin ratio, to assess not only different fat deposits but also fat dysfunction and its relationship with obesity-related comorbidities [55].

To strengthen generalizability and provide more comprehensive insights, future studies should include larger and more diverse populations, with a particular emphasis on increasing the representation of women across multiple centers. Regarding exercise, future intensive lifestyle intervention programs should incorporate structured training regimens aimed at improving body composition and preventing muscle mass and strength loss. Additionally, it will be essential to use objective measures of exercise adherence and to monitor muscle strength.

## 5. Conclusions

A hypocaloric pattern based on the Mediterranean diet resulted in a reduction of 8.2% in body weight at 12 months, together with a more favorable body composition profile and a reduction in VATI, SATI, and tendency in IMATI. These changes led to healthier metabolic and inflammatory parameters. These results highlight the need to incorporate weight loss programs in the treatment of patients with severe OSA undergoing CPAP treatment with the aim of improving their general health status. Further research is needed to determine the best OSA patient-centered strategies focused not only on weight loss but also on improving body fat distribution and particularly on reducing VAT and IMAT while preserving SM.

## Figures and Tables

**Table 1:** Characteristics of the study population at baseline.

	CG N = 16	IG N = 18	*p* Value
Age, years	52.0 [45.0; 54.0]	49.5 [46.2; 52.8]	0.377
Gender, Males (%)	15.0 (93.8)	15.0 (83.3)	0.604
Ethnicity, Causasian, n (%)	16 (100)	18 (100)	
Smokers, n (%)	3.0 (18.8)	2.0 (11.1)	0.648
Total IAH, events/h	69.0 (15.4)	69.8 (23.9)	0.904
Weight, Kg	106.0 (8.8)	99.5 (10.7)	0.073
BMI, Kg/m^2^	35.4 (2.9)	34.5 (2.6)	0.343
Neck Circumference, cm	43.9 (2.4)	42.5 (2.5)	0.113
Waist Circumference, cm	118.0 (8.6)	109.0 (7.4)	0.006 *
Estimated Fat Free Mass, Kg	33.9 [32.7; 34.8]	32.7 [31.3; 35.0]	0.509
Estimated Fat Mass, Kg	46.3 (8.3)	41.0 (6.9)	0.065
SM, cm^2^	191.0 [184.0; 197.0]	184.0 [176.0; 198.0]	0.509
VAT, cm^2^	274.0 (89.8)	225.0 (68.1)	0.096
SAT, cm^2^	323.0 (89.0)	301.0 (111.0)	0.537
TAT, cm^2^	618.0 (122.0)	541.0 (102.0)	0.065
IMAT, cm^2^	17.9 [14.7; 22.7]	14.1 [10.7; 18.4]	0.109
Muscle Attenuation, HU	43.9 [38.9; 45.7]	45.4 [42.6; 46.0]	0.336
SMI, cm^2^/m^2^	62.6 (8.1)	63.5 (7.4)	0.757
VATI, cm^2^/m^2^	92.9 (32.6)	77.2 (19.4)	0.118
SATI, cm^2^/m^2^	112.0 [80.1; 127.0]	97.5 [73.8; 128.0]	0.610
TATI, cm^2^/m^2^	209.0 (48.2)	189.0 (40.9)	0.215
IMATI cm^2^/m^2^	6.2 [4.9; 7.9]	5.3 [3.4; 7.3]	0.126

Values are expressed as mean (standard deviation), median [Q1; Q3], or n (%) where indicated. Significant values *p* < 0.05 are indicated by an asterisk. CG: control group; IG: intervention group; IAH: Index Apnea-Hypopnea; BMI: body mass index; SM: skeletal muscle; VAT: visceral adipose tissue; SAT: subcutaneous adipose tissue; TAT: total adipose tissue; IMAT: intramuscular adipose tissue; HU: Hounsfield unit; SMI: skeletal muscle index; VATI: visceral adipose tissue index; SATI: subcutaneous adipose tissue index; TATI: total adipose tissue index; IMATI: intramuscular adipose tissue index.

**Table 2:** Changes in macronutrient intake between baseline, 3, and 12 months.

	Baseline			Changes at 3 Months Compared to Baseline			Changes at 12 Months Compared to Baseline		
	CG N = 16	IG N = 18	*p*-Value	CG N = 16	IG N = 18	*p*-Value	CG N = 16	IG N = 18	*p*-Value
Energy (Kcal)	2790.0 (781.0)	2525.0 (784.0)	0.332	5.7 (868.0)	−1003.1 (856.0)	0.002 *	332.0 (904.0)	−532.9 (779.0)	0.006 *
Carbohydrates (g)	277.0 (87.5)	249.0 (104.0)	0.402	−37.3 (88.9)	−95.9 (121.3)	0.129	23.7 [−0.7; 50.8]	−42.1 [−121.0; 22.1]	0.040 *
Protein (g)	129.0 (58.0)	112.0 (42.9)	0.340	−8.2 (45.9)	−18.3 (43.5)	0.372	8.7 (48.7)	−7.1 (61.6)	0.419
Total Fat (g)	125.0 (45.1)	116.0 (41.7)	0.555	22.7 (65.0)	−56.4 (42.5)	<0.001 *	29.8 (54.6)	−19.7 (47.1)	0.009 *
Saturated Fat (g)	35.3 [26.8; 61.8]	29.8 [24.0; 46.0]	0.448	0.3 [−12.1; 14.5]	−18.7 [−23.7; −9.5]	0.033 *	8.1 [−0.4; 20.9]	−9.0 [20.2; 0.0]	0.042 *
Polinsaturated Fat (g)	22.0 [13.9; 26.5]	17.7 [11.3; 27.5]	0.756	−0.2 [−7.9; 5.7]	−5.8 [−19.2 –1.5]	0.044 *	−0.8 [−4.8; 10.0]	−4.1 [−8.3; 0.9]	0.121
Monoinsaturated Fat (g)	63.7 [49.5; 73.5]	53.9 [43.2; 65.0]	0.334	2.0 [−7.8; 30.5]	−23.7 [−38.7; −3.1]	0.011 *	10.4 [−2.3; 23.0]	−10.2 [−17.8; 7.6]	0.021 *
Fiber (g)	21.4 [15.7; 25.9]	18.2 [13.7; 21.1]	0.178	−1.4 (14.1)	3.1 (9.4)	0.298	1.7 [−10.0; 11.0]	−1.1 [−4.4; 3.5]	0.885

Values are expressed as mean (standard deviation). Significant *p* < 0.05 are indicated by an asterisk. CG: control group; IG: intervention group.

**Table 3:** Metabolic changes between baseline–3 months and baseline–12 months.

	3 Months Compared to Baseline			12 Months Compared to Baseline		
	CG N = 16	IG N = 18	*p*-Value	CG N = 16	IG N = 18	*p*-Value
Glucose, mmol/L	0.0 [−0.4; 0.2]	−0.5 [−0.7; −0.0]	0.047 *	−0.05 [−0.4; 0.4]	−0.4 [−0.6; 0.1]	0.360
HbA_1c_,%	−0.1 [−0.2; 0.0]	−0.3 [−0.4; −0.1]	0.155	−0.1 [−0.2; 0.1]	−0.2 [−0.3; −0.1]	0.031 *
Triglycerides, mmol/L	0.05 (0.7)	−0.3 (0.4)	0.143	−0.09 (0.9)	−0.3 (0.3)	0.396
Total Cholesterol, mmol/L	−0.3 (0.7)	−0.6 (0.7)	0.279	−0.4 (1.2)	−0.2 (0.8)	0.587
LDL C, mmol/L	−0.2 (0.7)	−0.6 (0.8)	0.177	−0.5 (1.4)	−0.3 (0.8)	0.633
HDL C, mmol/L	−0.07 (0.4)	0.05 (0.3)	0.345	−0.02 (0.2)	0.2 (0.3)	0.027 *
CRP, mg/L	0.0 [−1.0; 0.0]	−1.0 [−1.8; 0.0]	0.440	0 [−1.0;1.0]	−1 [−2.0;−0.3]	0.013 *
Systolic blood pressure, mmHg	0.3 (9.8)	−3.8 (22.0)	0.482	−5.0 (21.1)	−9.4 (21.0)	0.558
Diastolic blood pressure, mmHg	0.5 (12.7)	−4.8 (11.4)	0.215	−1.7 (15.8)	−5.9 (12.0)	0.400

Values are expressed as mean (standard deviation), median [Q1; Q3], or n (%) where indicated. Significant *p* < 0.05 are indicated by an asterisk. CG: control group; IG: intervention group; HbA_1c_: glycated hemoglobin; HDL C; high density lipoprotein cholesterol; LDL C: low density lipoprotein cholesterol; CRP: C-reactive protein.

**Table 4:** Mixed-effects models estimating weight change over time adjusted by age, baseline BMI, and baseline waist.

	Model 1: Adjusted by Treatment and Interaction Between Treatment and Visit			Model 2: Adjusted by ∆Kcal Intake			Model 3: Adjusted by ∆Lipid (g) Intake		
	B	CI	*p* Value	B	CI	*p* Value	B	CI	*p* Value
(Intercept)	104.5	100.1 to 108.8	<0.001 *	102.4	99.15 to 105.63	<0.001 *	102.4	99.16 to 105.6	<0.001 *
IG	−3.90	−10.0 to 2.22	0.208						
3-month visit	−2.31	−4.61 to −0.01	0.049 *	−5.34	−7.24 to −3.43	<0.001 *	−8.17	−10.58 to −5.77	<0.001 *
12-month visit	−0.12	−2.42 to 2.11	0.918	−4.57	−6.39 to −2.76	0.899	−2.97	−5.10 to −0.83	0.007 *
Age	−3.22	−6.03 to −0.42	0.025 *	−3.01	−6.16 to 0.13	0.060	−2.97	−6.11 to 0.16	0.063
Baseline waist	2.49	0.26 to 5.52	0.099	3.46	0.26 to 6.66	0.034 *	3.46	0.27 to 6.65	0.034 *
Baseline BMI	3.68	1.81 to 6.56	0.013 *	4.23	1.00 to 7.46	0.011 *	3.97	0.75 to 7.19	0.016 *
Treatment: 3-month visit	−7.31	−10.51 to −4.12	<0.001 *						
Treatment: 12-month visit	−8.11	−11.27 to −4.95	<0.001 *						
3-month ∆ (Kcal)				1.97	0.56 to 3.37	0.007 *			
12-month ∆(Kcal)				1.32	0.32 to 3.79	0.002 *			
3-month ∆ Lipids							2.14	0.44 to 3.74	0.009 *
12-month ∆ Lipids							3.01	0.60 to 5.43	0.015 *
Intraclass correlation coefficient = 0.84				Intraclass correlation coefficient = 0.84			Intraclass correlation coefficient = 0.84		

IG, intervention group; CI, confidence interval; BMI, body mass index. Significant *p* < 0.05 are indicated by an asterisk.

## Data Availability

The data presented in this study are available on request from the corresponding author.

## Supplementary Materials

The following supporting information can be downloaded at https://www.mdpi.com/article/10.3390/nu16244255/s1, Table S1:Patient baseline comorbidities and alcohol and smoking habits. Table S2. Baseline patients’ distribution of calories in macronutrients, sodijm and calcium. Table S3. Baseline metabolic parameters variables. Table S4. Patients’ respiratory changes between baseline–3 months and baseline–12 months. Table S5. Patients’ distribution of calories in macronutrients changes between baseline–3 months and baseline–12 months. Table S6: Linear model estimating IMAT at 12 months adjusted by age, baseline BMI, baseline waist, baseline IMAT and Kcal intake change.
