# Supplementary material for: Effects of a One-Year Intensified Weight Loss Program on Body Composition Parameters in Patients with Severe Obesity and Obstructive Sleep Apnea (OSA): A Randomized Controlled Trial
Source: Nutrients. 2024 Dec 10;16(24):4255. doi: 10.3390/nu16244255 (PMC11679847; doi:10.3390/nu16244255)
Supplement: Supplementary file 1 [file nutrients-16-04255-s001.zip › nutrients-3339644-supplementary.pdf]

ANNEX (ONLINE)

Table S1. Patient baseline comorbidities and alcohol and smoking habits.

	[ALL]<br>*N=34*	CG<br>*N=16*	IG<br>*N=18*	*P value*
**Hypertension, n (%)**	12.0 (35.3)	6.0 (37.5)	6.0 (33.3)	1.000
**Diabetes mellitus, n (%)**	3.0 (8.8)	3.0 (18.8)	0 (0.0)	0.094
**Dyslipidaemia, n (%)**	8.0 (23.5)	4.0 (25.0)	4.0 (22.2)	1.000
**Stroke, n (%)**	1.0 (2.9)	1.0 (6.3)	0.0 (0.0)	0.471
**Ischemic heart disease, n (%)**	2.0 (5.9)	0.0 (0.0)	2.0 (11.1)	0.487
**Hypothyroidism, n (%)**	1.0 (2.9)	1.0 (6.3)	0.0 (0.0)	0.471
**Alcohol, g**	8.0 [7.5; 22.2]	26.5 [10.5; 38.8]	7.0 [2.3; 8.0]	0.022*

Values are expressed as n (%) or as mean (standard deviation). Significant p <0.05 are indicated by an asterisk. CG: control group; IG: intervention group.

Table S2. Baseline patients' distribution of calories in macronutrients, sodijm and calcium.

	[ALL]<br>*N=34*	CG<br>*N=16*	IG<br>*N=18*	*P value*
**Carbohydrates, %**	41.2 [35.5; 46.4]	40.6 [36.8; 43.8]	41.9 [35.1; 46.4]	0.918
**Protein, %**	16.2 [13.5; 21.8]	16.3 [14.3; 21.8]	15.9 [13.1; 21.8]	0.666
**Total Fat, %**	40.5 (5.6)	39.9 (6.2)	41.1 (5.2)	0.574
**Saturated Fat, %**	11.2 [9.7; 16.2]	10.9 [9.6;16.8]	12.2 [9.8; 14.6]	0.931
**Polinsaturated Fat, %**	6.0 [4.5; 9.0]	6.2 [4.4;9.2]	5.9 [4.7; 7.8]	0.717
**Monoinsaturated Fat, %**	19.8 [17.0; 21.9]	19.6 [16.9; 22.6]	19.8 [19.0; 21.6]	0.629
**Sodium, mg**	2850.0 [2176.0; 4144.0]	3154.0 [2310.0; 4908.0]	2730.0 [2093.0; 4076.0]	0.569
**Calcium, mg**	879.0 [641.0; 1060.0]	846.0 [634.0; 1101.0]	913.0 [662.0; 1038.0]	1.000

Values are expressed as mean (standard deviation), median [Q1; Q3]. Significant p <0.05 are indicated by an asterisk. CG: control group; IG: intervention group.

Table S3. Baseline metabolic parameters variables

	[ALL]	CG	IG	*P value*
	N=34	*N=16*	*N=18*	
**Glucose**, mmol/L	5.8 [5.4; 6.2]	6.0 [5.5; 6.5]	5.6 [5.3; 6.0]	0.057
**HbA1c**, %	5.6 [5.4; 5.9]	5.6 [5.4; 5.9]	5.5 [5.4; 5.9]	0.425
**Triglycerides**, mmol/L	1.7 (0.6)	1.8 (0.7)	1.5 (0.6)	0.154
**Total cholesterol,** mmol/L	5.3 (0.9)	5.5 (0.8)	5.1 (1.0)	0.204
**LDL-C,** mmol/L	3.5 (1.1)	3.6 (1.1)	3.3 (1.2)	0.441
**HDL-C**, mmol/L	1.2 (0.3)	1.3 (0.3)	1.1 (0.3)	0.175
**CRP,** mg/L	3.0 [1.0; 4.0]	2.0 [1.0;4.0]	3.0 [2.0; 4.8]	0.316

Values are expressed as mean (standard deviation), median [Q1; Q3]. Significant p <0.05 are indicated by an asterisk. CG: control group; IG: intervention group; HbA_{1c}: glycated hemoglobin; LDL C; high density lipoprotein cholesterol; LDL C: low density lipoprotein cholesterol; CRP: C-reactive protein.

Table S4. Patients' respiratory changes between baseline – 3 months and baseline – 12 months.

	Baseline - 3 months				*Baseline - 12 months*			
	**[ALL]** *N=34*	**CG** *N=16*	**IG** *N=18*	***P value***	**[ALL]** *N=34*	**CG** *N=16*	**IG** *N=18*	***P value***
**Total AHI, events/h**	-16.8 (21.9)	-9.0 (25.2)	-23.7 (16.1)	0.056	-16.7 (20.2)	-13.5 (24.8)	-17.1 (15.3)	0.619
**Time with SpO_2<90, %**	-2.5 [-10.0; 1.0]	1.0 [-6.3; 3.0]	-5.0 [-11.5; -2.0]	0.047*	-1.5 [-6.5; 3.5]	-1.00 [-8.0; 6.0]	-3.0 [-5.0; 0.8]	0.417

Values are expressed as mean (standard deviation), median [Q1; Q3]. Significant p <0.05 are indicated by an asterisk. CG: control group; IG: intervention group; AHI: Apnea-Hypopnea index.

Table S5. Patients' distribution of calories in macronutrients changes between baseline – 3 months and baseline – 12 months.

	Baseline - 3 months				*Baseline - 12 months*			
	**[ALL]** *N=34*	**CG** *N=16*	**IG** *N=18*	***P value***	**[ALL]** *N=34*	**CG** *N=16*	**IG** *N=18*	***P value***
**Carbohydrates, %**	-1.8 (12.4)	-5.1 (10.1)	1.6 (13.8)	0.126	-2.0 (8.65)	-2.8 (5.6)	-1.3 (10.9)	0.614
**Protein, %**	3.1 (8.1)	-0.4 (6.3)	6.7 (8.2)	0.010*	1.5 [-1.9; 4.0]	-0.3 [-2.3; 2.7]	1.6 [-1.9; 9.1]	0.221
**Total Fat, %**	-0.1 (12.1)	6.6 (10.2)	-6.8 (10.1)	0.001*	3.4 (9.7)	4.5 (7.8)	2.4 (11.4)	0.540
**Saturated Fat, %**	-2.2 [-4.2; 0.9]	0.4 [-2.5;3.8]	-3.9 [-4.3;-2.2]	0.053	-0.7 [-4.4; 2.6]	1.0 [-0.8;3.1]	-3.2 [-6.0; 0.8]	0.031*
**Polinsaturated Fat, %**	-0.2 [-2.5; 0.9]	0.2 [-2.6; 1.7]	-0.6 [-2.5; 0.4]	0.343	0.0 [-2.4; 1.5]	-0.1 [-1.7; 2.1]	0.1 [-2.4; 0.8]	0.666
**Monoinsaturated Fat, %**	-0.6 [-2.9; 4.9]	0.4 [-1.6; 6.4]	-1.7 [-5.0; 2.8]	0.105	1.5 [-0.9; 4.7]	2.7 [-0.5; 3.5]	0.9 [-0.9; 4.7]	0.482
**Sodium, mg**	-59.8 [-129.5; 152.0]	-102.8 [-221.6; 101.0]	-18.0 [-86.8; 249.0]	0.163	7.5 [-221.5; 268.0]	3.8 [-169.6; 317.0]	17.6 [-221.5; 188.0]	0.614
**Calcium, mg**	-329.5 [-1443.7; 362.0]	293.0 [-524.4; 810.0]	-669.7 [-1643.8; -258.5]	0.013*	-196.8 (1811.0)	315.0 (1860.0)	-678.8 (1675.0)	0.118

Values are expressed as mean (standard deviation), median [Q1; Q3]. Significant p <0.05 are indicated by an asterisk. CG: control group; IG: intervention group.

Table S6: Linear model estimating IMAT at 12 months adjusted by age, baseline BMI, baseline waist, baseline IMAT and Kcal intake change

	**IMAT Model**		
	B	CI	P value
(Intercept)	16.76	15.6 to 17.9	<0.001*
IMAT	8.11	6.76 to 9.24	<0.001*
Age	8.11	-2.03 to 0.173	0.173
Baseline waist	0.28	-1.04 to 1.59	0.671
Baseline BMI	0.70	-0.64 to 2.04	0.462
*Δ*kcal 12 month visit-Baseline visit	1.41	0.24 to 2.58	0.020*

CI, confidence interval; IMAT, Intramuscular Adipose Tissue. Significant p <0.05 are indicated by an asterisk.
